# Supplementary material for: A Systematic Review Protocol of Opportunities for Noncommunicable Disease Prevention via Public Space Initiatives in African Cities
Source: Int J Environ Res Public Health. 2022 Feb 17;19(4):2285. doi: 10.3390/ijerph19042285 (PMC8872183; doi:10.3390/ijerph19042285)
Supplement: Supplementary file 1 [file ijerph-19-02285-s001.zip › ijerph-1457775-supplementary.pdf]

**Supplemetary**

**Pilot searches**

Search terms were created in consultation with a librarian as well as team members in multiple sites – England, Cameroon and Nigeria primarily. Searches were carried out on PubMed and translated for Scopus, Global Health and Web of Science to offer good breadth on literature on the intersection of public spaces and health in Africa. An initial search was run in May 2020 with a hit of 11,773 articles. From this initial term, search terms were refined to remove inapplicable MeSH terms from the PubMed search with particular reference to aspects of physical health, mental health and public spaces. Additionally, more MeSH terms were added to capture a wider range of public spaces and health outcomes. The final search was run in June 2020 and yielded 2839 articles. After these terms were agreed upon by the study team, they were translated for Web of Science, Global Health and Scopus.

**Table S1: Search strategy for PubMed**

| **Exposure** | **Outcome** |
|---|---|
| (((("coastline"[All Fields] OR "coastlines"[All Fields]) OR ("coast"[All Fields] OR "coasts"[All Fields])) OR ("countryside"[All Fields] OR "countrysides"[All Fields])) OR ("pavement"[All Fields] OR "pavements"[All Fields])) OR (((((((((((((((((((((((((((((((((((((((((((((((((((((((((((("Hills"[All Fields] OR "arena"[All Fields]) OR ("car"[All Fields] AND "Park"[All Fields])) OR "bridge"[All Fields]) OR (((("swimming pools"[MeSH Terms] OR ("swimming"[All Fields] AND "pools"[All Fields])) OR "swimming pools"[All Fields]) OR ("swimming"[All Fields] AND "pool"[All Fields])) OR "swimming pool"[All Fields])) OR (("lakes"[MeSH Terms] OR "lakes"[All Fields]) OR "lake"[All Fields])) OR "junction"[All Fields]) OR "junctions"[All Fields]) OR | ((((((((((((((((((((((((((((((((((((((((((((((((((((((((((((((("exercise"[MeSH Terms] OR "exercise"[All Fields]) OR "aerobics"[All Fields]) OR ("running"[MeSH Terms] OR "running"[All Fields])) OR "cricket"[All Fields]) OR ("swimming"[MeSH Terms] OR "swimming"[All Fields])) OR (("roller"[All Fields] OR "rollers"[All Fields]) AND ("skating"[MeSH Terms] OR "skating"[All Fields]))) OR ("safety"[MeSH Terms] OR "safety"[All Fields])) OR ("violence"[MeSH Terms] OR "violence"[All Fields])) OR (("mental health"[MeSH Terms] OR ("mental"[All Fields] AND "health"[All Fields])) OR "mental health"[All Fields])) OR "physical"[All Fields]) AND ("health"[MeSH Terms] OR "health"[All Fields])) OR (("physical fitness"[MeSH Terms] OR ("physical"[All Fields] AND "fitness"[All Fields])) OR "physical fitness"[All Fields])) OR (("men's health"[MeSH Terms] OR ("men's"[All |

| | |
|---|---|
| ("stadium"[All Fields] OR "stadiums"[All Fields])) OR ("roadside"[All Fields] OR "roadsides"[All Fields])) OR ("Vacant"[All Fields] AND "plots"[All Fields])) OR ("gardens"[MeSH Terms] OR "gardens"[All Fields])) OR ("outdoor"[All Fields] OR "outdoors"[All Fields])) OR "land"[All Fields]) OR "waterway"[All Fields]) OR "waterways"[All Fields]) OR ("neighbourhood"[All Fields] OR "neighborhood"[All Fields])) AND "playground"[All Fields]) OR "playgrounds"[All Fields]) OR "field"[All Fields]) OR "fields"[All Fields]) OR "Park"[All Fields]) OR ((("built"[All Fields] AND "environment"[All Fields]) OR "built environment"[All Fields]) OR "built environment"[MeSH Terms])) OR (("social environment"[MeSH Terms] OR ("social"[All Fields] AND "environment"[All Fields])) OR "social | Fields] AND "health"[All Fields])) OR "men's health"[All Fields])) OR (("women's health"[MeSH Terms] OR ("women's"[All Fields] AND "health"[All Fields])) OR "women's health"[All Fields])) OR (("population health"[MeSH Terms] OR ("population"[All Fields] AND "health"[All Fields])) OR "population health"[All Fields])) OR (((("air pollution"[MeSH Terms] OR ("air"[All Fields] AND "pollution"[All Fields])) OR "air pollution"[All Fields]) OR ("air"[All Fields] AND "quality"[All Fields])) OR "air quality"[All Fields])) OR ("yoga"[MeSH Terms] OR "yoga"[All Fields])) OR (("social capital"[MeSH Terms] OR ("Social"[All Fields] AND "capital"[All Fields])) OR "social capital"[All Fields])) OR ("gardening"[MeSH Terms] OR "gardening"[All Fields])) OR (((("water sports"[MeSH Terms] OR ("water"[All Fields] AND "sports"[All Fields])) OR "water sports"[All Fields]) OR ("water"[All Fields] AND "polo"[All Fields])) OR "water |

| | |
|---|---|
| environment"[All Fields])) OR ("public"[All Fields] AND "spaces"[All Fields])) OR ("green"[All Fields] AND "spaces"[All Fields])) OR ("blue"[All Fields] AND "space"[All Fields])) OR (((((("fitness centers"[MeSH Terms] OR ("fitness"[All Fields] AND "centers"[All Fields])) OR "fitness centers"[All Fields]) OR ("fitness"[All Fields] AND "centres"[All Fields])) OR "fitness centres"[All Fields]) OR ("health"[All Fields] AND "club"[All Fields])) OR "health club"[All Fields])) OR ((("community networks"[MeSH Terms] OR ("community"[All Fields] AND "networks"[All Fields])) OR "community networks"[All Fields]) OR "gym"[All Fields])) OR ("religious"[All Fields] AND ("groups"[All Fields] OR "group"[All Fields]))) OR ("temple"[All Fields] OR "temples"[All Fields])) OR ("mosque"[All Fields] OR "mosques"[All Fields])) OR "church"[All Fields]) OR "churches"[All | polo"[All Fields])) OR ("volleyball"[MeSH Terms] OR "volleyball"[All Fields])) OR ("football"[MeSH Terms] OR "football"[All Fields])) OR ("obesity"[MeSH Terms] OR "obesity"[All Fields])) OR ("Social"[All Fields] AND "connectedness"[All Fields])) OR ("Social"[All Fields] AND "connection"[All Fields])) OR (("track and field"[MeSH Terms] OR ("track"[All Fields] AND "field"[All Fields])) OR "track and field"[All Fields])) OR (("recreation"[MeSH Terms] OR "recreation"[All Fields]) OR "recreational"[All Fields])) OR ("tennis"[MeSH Terms] OR "tennis"[All Fields])) OR ((("racquet sports"[MeSH Terms] OR ("racquet"[All Fields] AND "sports"[All Fields])) OR "racquet"[All Fields]) AND ((("racquet sports"[MeSH Terms] OR ("racquet"[All Fields] AND "sports"[All Fields])) OR "racquet sports"[All Fields]) OR "squash"[All Fields]))) OR (("basketball"[MeSH Terms] OR "basketball"[All Fields]) OR "netball"[All Fields])) OR ("boxing"[MeSH |

| | |
|---|---|
| Fields]) OR "faith based organization"[All Fields]) OR "place of worship"[All Fields]) OR "roads"[All Fields]) OR ("sidewalk"[All Fields] OR "sidewalks"[All Fields])) OR ((((("bicycling"[MeSH Terms] OR "bicycling"[All Fields]) OR "bike"[All Fields]) AND "lanes"[All Fields]) OR "cycling"[All Fields]) OR "cycle"[All Fields]) AND "lanes"[All Fields])) OR "market"[All Fields]) OR "markets"[All Fields]) OR "marketplace"[All Fields]) OR "marketplaces"[All Fields]) OR (((("shopping"[All Fields] OR "shops"[All Fields]) AND "Or"[All Fields]) AND "shop"[All Fields]) AND ("arcade"[All Fields] OR "arcades"[All Fields]))) OR "mall"[All Fields]) OR "plaza"[All Fields]) OR "plazas"[All Fields]) OR "Park"[All Fields]) OR "parkland"[All Fields]) OR "parklands"[All Fields]) OR ("rivers"[MeSH Terms] OR "rivers"[All Fields])) OR "square"[All Fields]) OR | Terms] OR "boxing"[All Fields])) OR ("Social"[All Fields] AND "cohesion"[All Fields])) OR (("health promotion"[MeSH Terms] OR ("health"[All Fields] AND "promotion"[All Fields])) OR "health promotion"[All Fields])) OR "wellbeing"[All Fields]) OR ("soccer"[MeSH Terms] OR "soccer"[All Fields])) OR "bicycle"[All Fields]) OR "bicycles"[All Fields]) OR ("bicycling"[MeSH Terms] OR "bicycling"[All Fields])) OR ("baseball"[MeSH Terms] OR "baseball"[All Fields])) OR "athletics"[All Fields]) OR "athletic"[All Fields]) OR (("sports"[MeSH Terms] OR "sports"[All Fields]) OR "sporting"[All Fields])) OR (("leisure activities"[MeSH Terms] OR ("leisure"[All Fields] AND "activities"[All Fields])) OR "leisure activities"[All Fields])) OR "participation"[All Fields]) OR "injury"[All Fields]) OR "accidents"[MeSH Terms]) OR ("Social"[All Fields] AND "activities"[All Fields])) OR ("Social"[All Fields] AND "activity"[All Fields])) OR |

| | |
|---|---|
| "squares"[All Fields]) OR "townhall"[All Fields]) OR "community"[All Fields]) AND "hall"[All Fields]) OR "street"[All Fields]) OR "streets"[All Fields]) OR "beach"[All Fields]) OR "beaches"[All Fields]) OR ("recreational"[All Fields] AND ("center"[All Fields] OR "centre"[All Fields]))) | "inclusion"[All Fields]) OR (("sports"[MeSH Terms] OR "sports"[All Fields]) OR "sport"[All Fields])) OR ((("play and playthings"[MeSH Terms] OR ("play"[All Fields] AND "playthings"[All Fields])) OR "play and playthings"[All Fields]) OR "play"[All Fields])) OR ("relaxation"[MeSH Terms] OR "relaxation"[All Fields])) OR (("hobbies"[MeSH Terms] OR "hobbies"[All Fields]) OR "hobby"[All Fields])) OR (("dancing"[MeSH Terms] OR "dancing"[All Fields]) OR "dance"[All Fields])) OR ((("leisure activities"[MeSH Terms] OR ("leisure"[All Fields] AND "activities"[All Fields])) OR "leisure activities"[All Fields]) OR "leisure"[All Fields])) OR "ball"[All Fields]) OR (("football"[MeSH Terms] OR "football"[All Fields]) OR "rugby"[All Fields])) OR ((("exercise"[MeSH Terms] OR "exercise"[All Fields]) OR ("physical"[All Fields] AND "exercise"[All Fields])) OR "physical exercise"[All Fields])) OR ((("public health"[MeSH Terms] OR ("public"[All Fields] AND |

| | |
|---|---|
| | "health"[All Fields])) OR "public health"[All Fields]) OR "health"[MeSH Terms])) OR ("health"[All Fields] AND ("outcome"[All Fields] OR "outcomes"[All Fields]))) OR "pollution"[All Fields]) OR ((("physical"[All Fields] AND "activities"[All Fields]) OR "activity"[All Fields]) OR "physical activity"[All Fields])) OR "cycling"[All Fields]) OR (("walking"[MeSH Terms] OR "walking"[All Fields]) OR "games"[All Fields]) |

| | |
|---|---|
| **Population** "ethiopia"[MeSH Terms] OR "ethiopia"[All Fields] OR "gabon"[MeSH Terms] OR "gabon"[All Fields] OR "gambia"[MeSH Terms] OR "gambia"[All Fields] OR "ghana"[MeSH Terms] OR "ghana"[All Fields] OR "guinea"[MeSH Terms] OR "guinea"[All Fields] OR "equatorial guinea"[All Fields] OR "guinea-bissau"[MeSH Terms] OR "guinea-bissau"[All Fields] OR "kenya"[MeSH Terms] OR "kenya"[All Fields] OR "lesotho"[MeSH Terms] OR "lesotho"[All Fields] OR "liberia"[MeSH Terms] OR "liberia"[All Fields] OR "libya"[MeSH Terms] OR "libya"[All Fields] OR "madagascar"[MeSH Terms] OR "madagascar"[All Fields] OR "malawi"[MeSH Terms] OR "malawi"[All Fields] OR "mali"[MeSH Terms] OR "mali"[All Fields] OR | ((((((((((((((((("cities"[MeSH Terms] OR "cities"[All Fields]) OR "city"[All Fields]) OR "town"[All Fields]) OR "towns"[All Fields]) OR "urban"[All Fields]) OR "metropolitan"[All Fields]) OR "conurbation"[All Fields]) OR "municipality"[All Fields]) OR "municipalities"[All Fields]) OR "city planning"[MeSH Terms]) OR "urban planning"[All Fields]) OR "metro"[All Fields]) OR "metropolis"[All Fields]) OR ("megacities"[All Fields] OR "megacity"[All Fields])) OR "urban renewal"[MeSH Terms]) OR ("urban"[All Fields] AND "renewal"[All Fields])) OR "urban renewal"[All Fields]) OR (((((((((((((("urban"[All Fields] OR "urbanicity"[All Fields]) OR "urbanism"[All Fields]) OR "urbanity"[All Fields]) OR "urbanization"[MeSH Terms]) OR "urbanization"[All Fields]) OR "urbanize"[All Fields]) OR "urbanized"[All Fields]) OR "urbanizes"[All Fields]) OR |

| | |
|---|---|
| "mauritania"[MeSH Terms] OR "mauritania"[All Fields] OR "mauritius"[MeSH Terms] OR "mauritius"[All Fields] OR "morocco"[MeSH Terms] OR "morocco"[All Fields] OR "mozambique"[MeSH Terms] OR "mozambique"[All Fields] OR "namibia"[MeSH Terms] OR "namibia"[All Fields] OR "niger"[MeSH Terms] OR "niger"[All Fields] OR "nigeria"[MeSH Terms] OR "nigeria"[All Fields] OR "rwanda"[MeSH Terms] OR "rwanda"[All Fields] OR "sao tome and principe"[MeSH Terms] OR "sao tome and principe"[All Fields] OR "senegal"[MeSH Terms] OR "senegal"[All Fields] OR "seychelles"[MeSH Terms] OR "seychelles"[All Fields] OR "sierra leone"[MeSH Terms] OR "sierra leone"[All Fields] OR "somalia"[MeSH Terms] OR "somalia"[All Fields] OR | "urbanizing"[All Fields]) ) OR "urbanisation"[All Fields]) OR "urbanise"[All Fields]) OR "urbanised"[All Fields]) OR "urbanises"[All Fields]) OR "urbanising"[All Fields]) <br><br> **Total hits: 2839 articles** |

| | |
|---|---|
| "south africa"[MeSH Terms] OR "south africa"[All Fields] OR "south sudan"[MeSH Terms] OR "south sudan"[All Fields] OR "sudan"[MeSH Terms] OR "sudan"[All Fields] OR "tanzania"[MeSH Terms] OR "tanzania"[All Fields] OR "togo"[MeSH Terms] OR "togo"[All Fields] OR "tunisia"[MeSH Terms] OR "tunisia"[All Fields] OR "uganda"[MeSH Terms] OR "uganda"[All Fields] OR "zambia"[MeSH Terms] OR "zambia"[All Fields] OR "zimbabwe"[MeSH Terms] OR "zimbabwe"[All Fields] OR "africa"[MeSH Terms] OR "africa"[All Fields] | |

Table S2: Search strategy for Web of Science

| Exposure | Outcome |
| --- | --- |
| (coast* OR country* OR pavement* OR hills OR arena OR carpark OR bridge OR swimming pool* OR lake* OR junction* OR stadium* OR roadside* OR vacant plot* OR garden* OR outdoor* OR land OR waterway* OR neighbourhood playground* OR neighborhood playground* OR playground* OR field* OR park OR built environment OR social environment OR public space* OR green space* OR blue space* OR fitness center* OR fitness centre* OR health club OR community network* OR gym OR religious group* OR temple* OR mosque* OR church* OR faith based organization OR place of worship OR road* OR sidewalk* OR bicycling OR bike lanes OR cycl* OR cycle lanes OR market* OR shop* OR arcade* OR mall OR plaza* OR park* OR river* OR square OR street* OR beach* OR recreational center OR recreational centre) | (Exercise OR Aerobics OR Running OR Cricket OR Swim* OR Roller skating OR Safety OR Violence OR mental health OR physical health OR physical fitness OR men's health OR women's health OR population health OR air pollution OR air quality OR yoga OR social capital OR garden* OR water sports OR water polo OR volleyball OR football OR obesity OR social connectedness OR social connection OR track and field OR recreation* OR tennis OR racquet sports OR basketball OR netball OR boxing OR social cohesion OR health promotion OR wellbeing OR soccer OR bicycle* OR athletics OR leisure activities OR participation OR injury OR accidents OR social activit* OR inclusion OR sport* OR play* OR relaxation OR hobb* R dance* OR leisure activities OR leisure OR ball |

| | |
|---|---|
| | OR football OR rugby OR exercise OR physical exercise OR public health OR health outcome OR pollution OR physical activit* OR cycling OR walking OR games) |
| Population<br><br>(cit* OR town* OR urban OR metropolitan OR conurbation OR municipalit* OR city planning OR urban planning OR metro* OR megacit* OR urban*) | (ethiopia OR Gabon OR Gambia OR Ghana OR Guinea OR Equatorial Guinea OR Guinea Bissau OR Kenya OR Lesotho OR Liberia OR Libya OR Madagascar OR Malawi OR Mali OR Mauritania OR Mauritius OR Morocco OR Mozambique OR Namibia OR Niger OR Nigeria OR Rwanda OR Sao Tome and Principe Or Senegal OR Seychelles OR Sierra Leone OR Somalia OR South Africa OR South Sudan OR Sudan OR Tanzania OR Togo OR Tunisia OR Uganda OR Zambia OR Zimbabwe OR Africa)<br><br>Total hits: 10799 |

Table S3: Search strategy for Global Health

| Exposure | Outcome |
| --- | --- |
| (coastline or coastlines or coast or coasts or countryside or countrysides or pavement or pavements or arena or hills or car park or bridge or swimming pools or swimming pool or lakes or lake or junction or junctions or stadium or stadiums or roadside or roadsides or vacant plots or gardens or outdoors or land or waterways or waterway or neighbourhood playground or neighbourhood playgrounds OR neighborhood playground OR neighborhood playgrounds or field or fields or park or built environment or social environment or public spaces or green spaces or blue space or fitness centers or fitness centres or health club or health club or community networks or gym or religious groups or religious group or temple or temples or | (exercise or aerobics or running or cricket or swimming or roller skating or safety or violence or mental health or physical health or physical fitness or men's health or women's health or population health or air pollution or air quality or yoga or social capital or gardening or water sports or water polo or volleyball or football or obesity or social connectedness or social connection or track and field or recreation or recreational tennis or racquet sports or squash or basketball or netball or boxing or social cohesion or health promotion or wellbeing or soccer or bicycle or bicycles or bicycling or baseball or athletics or sports or sporting or leisure activities or participation or injury or accidents or social activities or social activity or inclusion or sports or sport or play or plaything or relaxation or hobbies or hobby or dancing or dance or leisure activities or leisure or ball or football or rugby or exercise or physical |

| | |
|---|---|
| mosque or mosques or church or churches or faith based organization or place of worship or roads or sidewalk or sidewalks or bicycling or bike lands or cycling or cycle lanes or market or markets or marketplace or marketplaces or shops or shop or arcade or mall or plaza or plazas or park or parkland or parklands or rivers or square or squares or townhall or community hall or street or streets or beach or beaches or recreational center or recreational centre ) | exercise or public health or health or health outcome or health outcomes or pollution or physical activities or physical activity or cycling or walking or games ) |
| Population<br><br>( cities or city or town or towns or urban or metropolitan or conurbation or municipality or municipalities or city planning or urban planning or metro or metropolis or megacities or megacity or urban renewal or urban or urbanicity or urbanism or urbanity or urbanization | ( ethiopia or gabon or gambia or ghana or guinea or equatorial guinea or guinea-bissau or kenya or lesotho or liberia or libya or madagascar or malawi or mali or mauritania or mauritius or morocco or mozambique or namibia or niger or nigeria or rwanda or sao tome and principe or senegal or seychelles or |

| | |
|---|---|
| or urbanize or urbanized or urbanizes or urbanizing or urbanisation or urbanise or urbanised or urbanises or urbanising ) | sierra leone or somalia or south africa or south sudan or sudan or tanzania or togo or tunisia or uganda or zambia or zimbabwe or africa )<br><br>Total hits: 7514 |

Table S4: Search strategy for Scopus

| Exposure | Outcome |
|---|---|
| ( TITLE-ABS-KEY ( coast* OR country* OR pavement* OR arena OR "car park" OR bridge OR "swimming pool*" OR lake* OR junction* OR stadium OR roadside* OR "vacant plot" OR garden OR outdoor* OR land OR waterway* OR playground* OR neighbourhood OR neighborhood OR field* OR park OR "built environment" OR "social environment" OR "green space*" OR "blue space*" OR "public space*" OR "fitness centers" OR "fitness centres" OR "health club" OR "community network*" OR | AND ( TITLE-ABS-KEY ( exercise OR aerobics OR running OR cricket OR swimming OR "roller skating" OR safety OR violence OR "mental health" OR "physical health" OR "physical fitness" OR "men's health" OR "women's health" OR "population health" OR "air pollution" OR "air quality" OR yoga OR "social capital" OR gardening OR "water sports" OR "water polo" OR volleyball OR football OR obesity OR "social connectedness" OR "social connection" OR "track and field" OR receation* OR tennis OR "racquet sports" OR squash OR basketball OR netball OR |

| | |
|---|---|
| gym OR "religious group" OR temple* OR mosque* OR church* OR "faith based organization*" OR "place of worship" OR roads OR sidewalk* OR *cycling OR "cycle lanes" OR market* OR shop* OR arcade* OR mall OR plaza* OR park* OR river* OR square* OR townhall OR "community hall" OR street* OR beach* OR "recreational center" OR "recreational centre" ) ) | boxing OR "social cohesion" OR "health promotion" OR wellbeing OR soccer OR bicycl* OR baseball OR athletic* OR sport* OR "leisure activities" OR participation OR injury OR accidents OR "social activities" OR "social activity" OR inclusion OR sport* OR play* OR relaxation OR hobby OR hobbies OR danc* OR "leisure activities" OR leisure* OR ball OR football OR rugby OR exercise OR "physical exercise" OR "public health" OR "public outcomes" OR pollution OR "physical activities" OR "physical activity" OR cycling OR walking OR games ) ) |
| Population<br><br>( TITLE-ABS-KEY ( ethiopia OR gabon OR gambia OR ghana OR guinea OR "equatorial guinea" OR "guinea-bissau" OR kenya OR lesotho OR liberia OR libya OR | (TITLE-ABS-KEY ( city* OR cities* OR town* OR urban OR metropolitan OR conurbation OR municipalit* OR "city planning" OR "urban planning" OR metro* OR megacit* OR "urban renewal" OR urbani* ) ) AND ( LIMIT-TO ( PUBYEAR , 2020 ) OR LIMIT-TO ( PUBYEAR , 2019 ) |

| | |
|---|---|
| madagascar OR malawi OR mali OR mauritania OR mauritius OR morocco OR mozambique OR namibia OR niger OR nigeria OR rwanda OR "sao tome and principe" OR senegal OR seychelles OR "sierra leone" OR somalia OR "south Africa" OR "south sudan" OR sudan OR tanzania OR togo OR tunisia OR uganda OR zambia OR zimbabwe OR africa ) ) | OR LIMIT-TO ( PUBYEAR , 2018 ) OR LIMIT-TO ( PUBYEAR , 2017 ) OR LIMIT-TO ( PUBYEAR , 2016 ) OR LIMIT-TO ( PUBYEAR , 2015 ) OR LIMIT-TO ( PUBYEAR , 2014 ) OR LIMIT-TO ( PUBYEAR , 2013 ) OR LIMIT-TO ( PUBYEAR , 2012 ) OR LIMIT-TO ( PUBYEAR , 2011 ) OR LIMIT-TO ( PUBYEAR , 2010 ) OR LIMIT-TO ( PUBYEAR , 2009 ) OR LIMIT-TO ( PUBYEAR , 2008 ) OR LIMIT-TO ( PUBYEAR , 2007 ) OR LIMIT-TO ( PUBYEAR , 2006 ) OR LIMIT-TO ( PUBYEAR , 2005 ) OR LIMIT-TO ( PUBYEAR , 2004 ) OR LIMIT-TO ( PUBYEAR , 2003 ) OR LIMIT-TO ( PUBYEAR , 2002 ) OR LIMIT-TO ( PUBYEAR , 2001 ) OR LIMIT-TO ( PUBYEAR , 2000 ) OR LIMIT-TO ( PUBYEAR , 1999 ) OR LIMIT-TO ( PUBYEAR , 1998 ) OR LIMIT-TO ( PUBYEAR , 1997 ) OR LIMIT-TO ( PUBYEAR , 1996 ) OR LIMIT-TO ( PUBYEAR , 1995 ) OR LIMIT-TO ( |

| | |
|---|---|
| | PUBYEAR , 1994 ) OR LIMIT-TO ( PUBYEAR , 1993 ) OR LIMIT-TO ( PUBYEAR , 1992 ) OR LIMIT-TO ( PUBYEAR , 1991 ) OR LIMIT-TO ( PUBYEAR , 1990 ) ) AND ( LIMIT-TO ( DOCTYPE , "ar" ) ) AND ( LIMIT-TO ( LANGUAGE , "English" ) )<br><br>Total hits: 8991 |
